# The excess economic burden of mental disorders: findings from a cross-sectional prevalence survey in Austria

**DOI:** 10.1007/s10198-020-01200-0

**Published:** 2020-05-26

**Authors:** Agata Łaszewska, Johannes Wancata, Rebecca Jahn, Judit Simon

**Affiliations:** 1 Department of Health Economics, Center for Public Health, Medical University of Vienna, Kinderspitalgasse 15/I, 1090 Vienna, Austria; 2 Clinical Division of Social Psychiatry, Department of Psychiatry and Psychotherapy, Medical University of Vienna, Währinger Gürtel 18-20, 1090 Vienna, Austria

**Keywords:** Mental disorders, Excess cost, Economic burden, Productivity loss, C31 (Multiple variables: cross-sectional models), I1 (Health) or I18 (Health: government policy, regulation, public health), H51 (Government expenditures and health)

## Abstract

Information about the scope of mental disorders (MDs), resource use patterns in health and social care sectors and economic cost is crucial for adequate mental healthcare planning. This study provides the first representative estimates about the overall utilisation of resources by people with MDs and the excess healthcare and productivity loss costs associated with MDs in Austria. Data were collected in a cross-sectional survey conducted on a representative sample (*n* = 1008) between June 2015 and June 2016. Information on mental health diagnoses, 12-month health and social care use, medication use, comorbidities, informal care, early retirement, sick leave and unemployment was collected via face-to-face interviews. Generalised linear model was used to assess the excess cost of MDs. The healthcare cost was 37% higher (*p* = 0.06) and the total cost was twice as high (*p* < 0.001) for the respondents with MDs compared to those without MDs. Lost productivity cost was over 2.5-times higher (*p* < 0.001) for those with MDs. Participants with severe MDs had over 2.5-times higher health and social care cost (*p* < 0.001) and 9-times higher mental health services cost (*p* < 0.001), compared to those with non-severe MDs. The presence of two or more physical comorbidities was a statistically significant determinant of the total cost. Findings suggest that the overall excess economic burden on health and social care depends on the severity of MDs and the number of comorbidities. Both non-severe and severe MDs contribute to substantially higher loss productivity costs compared to no MDs. Future resource allocation and service planning should take this into consideration.

**Supplementary Information:**

The online version contains supplementary material available at 10.1007/s10198-020-01200-0.

## Introduction

The latest estimates about the prevalence of mental disorders (MDs) across the European Union (EU) countries indicate that 17% of the EU residents are affected by MDs each year [1]. It is expected that disability and societal burden of MDs will further increase, placing MDs at the top of the list of policy priorities [2]. Due to the relatively high number of people affected by MDs, the need for services to support them and the demand for cost information by decision makers in the area of psychiatric care have increased considerably [3].

Despite the increasing importance of evidence on the economic burden for use in priority setting, there is scarce evidence on the service utilisation patterns and costs associated with MDs in many countries including Austria. The “Cost of disorders of the brain in Europe” studies that attempted to calculate the costs associated with mental and neurological disorders for the European countries highlighted the importance of national estimates based on national-level data due to potentially differing epidemiological data and variability in the cost of MDs due to healthcare system differences [4]. In the case of Austria, due to the lack of national data, these estimates were derived by extrapolating available data from other countries rather than reflecting real national estimates based on the local healthcare system [5–7].

In Austria, 99.9% of population is covered by social health insurance (SHI) [8]. Currently, 18 SHI funds exist in Austria, however, ongoing reform of the social security system aims to reduce this number to five SHI funds [9]. In Austria, direct access to specialists in the outpatient sector, ambulatory hospital clinics and in some cases to inpatient care is common as there is no formal gate-keeping mechanism in place. Health spending amounts to around 10–11% of GDP [10], ranking Austria as the 10th most expensive system in the OECD countries in 2016 [11]. Among the EU countries, Austria is one of the leaders in terms of hospital beds per 1000 residents (7.4 per 1000 compared to the EU28 average of 5.1, in 2016) and has one of the highest number of practising doctors per 1000 residents (5.1 per 1000 compared to EU28 average of 3.6, in 2016) [1]. Funding and organisation of mental health services is fragmented and there is no separate budget allocated to mental health. Psychotherapy can be fully or partially covered by SHI [12]. Funding for other mental health services (e.g., psychosocial services and day centres) relies mostly on regional governments and varies considerably between federal states [8, 13].

Several studies examined economic and societal burden of different diagnoses of MDs in the general adult population in different countries [6, 14–20]. However, studies examining an excess cost of MDs are less common. Excess cost provides an estimates of the cost difference between populations with and without mental health diagnosis adjusted for existing comorbidities and socio-economic characteristics [21]. Such information is crucial for priority setting and service planning across diseases. Most of the existing studies examined the excess cost for individual mental health diagnoses, including schizophrenia or psychotic disorders [22–25], major or minor depression [26–28], anxiety disorders [29, 30], alcohol dependence [21], and bipolar disorder [31, 32]. Only a few studies looked at the excess cost of MDs overall in a general population sample, which is more relevant for health services organisation aspects considering that many services target MDs in general rather than one specific disorder. For example, Smit et al. (2006) examined excess cost of MDs in a general population in the Netherlands [33]. However, in their study, MDs with a 12-months prevalence below 1%—such as bipolar disorder, obsessive–compulsive disorder, eating disorders or schizophrenia—were excluded. There is no study that looked at the excess cost of MDs as a whole in a country with a similar healthcare system to Austria, i.e**.**, no formal gate-keeping mechanism and unrestricted access to all levels of care (primary, secondary and tertiary care). Besides providing the first reliable, representative national cost estimates in Austria, the current study also makes an important contribution to the European evidence landscape and mental health policy making.

## Materials and methods

### Study design

In Austria, due to the fragmented planning, financing and provision of healthcare among different institutions, including federal government, regional governments, SHI, and private or non-profit organisations, linked data between the different sectors or service levels, e.g., outpatient and inpatient services, is not available for study. Therefore, representative multi-sectoral information on the utilisation of services and resources can be collected only via general population surveys. For the current study, data were collected as part of the Austrian Psychiatric Prevalence Study (APPS), a cross-sectional nation-wide survey conducted on a representative sample of the non-elderly, non-institutionalised adult Austrian general population. Sampling procedure included a multistage cluster sampling based on geographical regions with stratification first at the federal state and then at the district levels and by sex. The sample was drawn across all federal states, with districts being selected in each state according to a random system. Subsequently, addresses were chosen for these selected districts using again a random system. The target sample size was 1000, primarily defined as suitable for the epidemiological components of the study. No formal power analysis was performed. Details about the sampling process and study design have been published elsewhere [34].

### Study participants

Adults aged 18–65, living in private households and with a sufficient knowledge of the German language were included. Children, adolescents and those above 65 years old, as well as people living in institutions or homeless, and those whose German knowledge was not sufficient to undergo an interview, were not considered.

### Data collection

Face-to-face personal interviews took place with each participant between June 2015 and June 2016. Participants received a remuneration of 20 EUR for participation.

Mental health diagnoses were assessed by professionals trained in psychiatry using the 10th version of “Present State Examination” (PSE-10), a valid and reliable standard instrument for the assessment and classification of psychiatric syndromes in adults developed by the World Health Organisation (WHO) [35–37]. For each respondent, 12-month prevalence of MDs classified according to the International Classification of Diseases (ICD-10) was reported based on the PSE-10. Mental health diagnoses considered in this study included: substance use disorders, schizophrenia, schizotypal and delusional disorders, mood disorders (mania, bipolar disorder, depression, other mood disorders), neurotic, stress-related and somatoform disorders (phobia, anxiety, obsessive–compulsive disorder, somatoform disorder, neurotic disorders), eating disorders and other behavioural syndromes, and personality disorders [38].

For the information about the physical comorbidities, the respondents were asked to indicate co-existing diseases (including chronic diseases) which they experienced in the last 12 months. Self-reported comorbidities were categorised in twenty-two disease groups (details in Online Resource Table 1).

Information on the utilisation of health and social care services was reported by the respondents for the recall period of the previous 12 months. Collected resource use items included outpatient non-mental health specific contacts (GP, specialists, non-physician specialists (ergotherapist, physiotherapist, nurse, social worker, life counselling, naturopath, other)); outpatient mental health specific contacts including psychiatrist, psychotherapist, psychologist, counselling centre, day centre, psychiatric day clinic, psychosocial care, and sheltered workshop; and inpatient stay.

Since some of the above listed services, especially mental health specific services are provided and financed from the local governments’ social care budget, the list of services specified above is henceforth referred to as ‘health and social care services’.

### Costing methods

Resource use items (e.g., outpatient visits, hospital length of stay) were multiplied by their national 2016 unit costs in EUR (€). The summary of unit costs used in the study is provided in Online Resource Table 2. Healthcare costs in the outpatient sector were calculated from the perspective of the SHI if reimbursement tariffs were available. Whenever reimbursement tariff was not available, e.g., for the services such as alternative medicine, the market price was considered. Cost results were calculated from both the health and social care, and societal perspectives.

Information on the ‘cost per day of inpatient care’ in different hospital departments was obtained from the Ministry of Health based on national-level administrative cost information on inpatient and day patient cases in year 2016.

To derive the cost of psychotherapy, a multiple-perspective approach similar to the one previously used by Zechmeister et al. [39] was adopted. In Austria, opportunity for full reimbursement of psychotherapy varies across the country [40]. Also, the level of partial reimbursement, in case a physician does not have a contract with an SHI, vary between € 21.80 and € 50.00 [41] for an individual therapy session with the remaining costs to be paid out-of-pocket by the patient [42]. In year 2016, the average patient co-payment was around €50 per session [8, 39]. Therefore, the unit cost per psychotherapy was calculated at €71.8 (€50 + €21.80).

In case of services for which the patient’s co-payment is substantial and/or which are largely regarded as private services and paid mostly out-of-pocket, i.e., ergotherapy, physiotherapy, psychologist, the actual market price was used [43, 44].

The cost of informal care was valued using the hourly wage for domestic help according to Drummond et al. [45]. Costs due to sick leave, early retirement and unemployment were calculated using the human capital approach based on the value of mean gross salary for full-time and part-time work in Austria [46].

Information on the type of medication, dose per day, duration and regularity of taking the medication over the past 12 months was retrieved from the survey. Social insurance medication prices were taken from the Austrian ‘Erstattungskodex’ [47]. Average cost per milligram was calculated and multiplied by the daily dose reported by the respondents. If the dose was not reported, the recommended daily dose was assumed. If information on the duration of taking the medication was missing, or if it was reported by the respondents that the medication was taken as per need, we assumed one package of the particular medication purchased in a given year as per Friemel et al. [48].

Unit cost information available only for another year was deflated or inflated to year 2016 based on health-specific inflation rate provided by Statistics Austria [49]. The time horizon of the analysis was limited to one year; therefore, correction for inflation and discounting was not applied.

### Analysis

Annual prevalence-based approach was used to estimate the excess economic burden attributable to MDs over a one-year time period. This is a common approach in studies assessing the annual economic burden of a health problem under investigation [50].

Characteristics of survey respondents were presented as means and standard deviations for continuous variables and frequencies and percentages for categorical variables. Comparison of the respondents with at least one MD and without MDs with regards to the demographic and socio-economic characteristics was explored using the chi-square test for categorical variables and the *t*-test for continuous variables. Distribution of resource use and costs was examined using histograms and quantile–quantile (*Q*–*Q*) plots. Due to right-skewed data, resource use information was compared using Wilcoxon rank sum (Mann–Whitney) test. The excess cost of MDs was estimated using generalised linear model (GLM) with gamma distribution and log link function [51]. Results of the GLM model were presented as exponentiated coefficients. Excess costs were calculated in EURO (€) from the GLM predictions for year 2016. Correctness of the link function was tested using a Box–Cox test [52], while correctness of the family (gamma) distribution was tested using Park test [51].

Because of the wide variety of MDs included in the study, another GLM regression was run comparing respondents with severe MDs vs. non-severe MDs. Mental health diagnoses were divided in severe and non-severe categories based on the type of diagnoses as defined in previous studies [53–55]. Schizophrenia, schizoaffective disorder, psychoses, bipolar disorder and major depressive disorder were classified as severe mental health conditions (severe MDs). The remaining diagnoses were classified as non-severe MDs. All GLM regressions were adjusted for sex, age, education level, and the number of physical comorbidities as categorical variables. A further exploratory disease-specific subgroup analysis by ICD-10 groups F1–F5 was also conducted.

For the calculation of the excess cost, missing values of cost data were imputed using Multiple Imputation by Chained Equations (MICE) with 50 imputations using categorical variables: a diagnosis of MD, sex, age group, place of living (number of inhabitants), number of physical comorbidities and current employment status as predictor variables. In all analyses in Stata these variable names were prefixed by “*i*.” to specify indicators for each level (category) of the variable. The distributions of variables before and after multiple imputation were inspected using summary statistics (mean, median, 25th and 75th percentiles) for the high cost variables (i.e., inpatient treatment) to make sure that multiple imputation has not influenced these distributions. Total resource use data were visually inspected for outliers using box plots.

Separate sensitivity analyses were conducted using (1) post-stratification weights accounting for any distributional differences in geographical areas, sex and age groups between the study sample and the general population in Austria [34], and (2) using complete cases. All analyses were performed in Stata 15.1 [56].

## Results

### Sample characteristics

A total of 3049 potential study participants were contacted via phone, of which 642 people could not be reached. Of those with whom a phone contact took place, 1203 persons declined to participate in the study and 169 could not be included for reasons of illness or lack of German language skills. A total of 1008 people (response rate 33%) participated in the survey. One respondent was excluded from all analyses due to implausible values of resource use reported which was not possible to track or verify. No further outliers had to be removed or adapted. All economic analyses were conducted on a sample size of 1007 respondents.

Differences in socio-demographic characteristics between the respondents with at least one MD vs. no MDs, as well as respondents with severe and non-severe MDs are presented in Table 1. Overall, 229 respondents had a diagnosis of at least one MD, of which 117 (51%) had severe MDs. Mean age was 47 years in the group of respondents with MDs and 45 years among respondents without MDs. There were more women among participants with diagnoses of MDs (56%), compared to those without MDs (49%). Compared to the population without MDs, people suffering from MDs were more likely to live in the bigger cities, be unemployed, belong to lower social class, and live alone. Respondents with severe and non-severe MDs did not differ significantly with respect to their socio-demographic characteristics except for ‘place of birth’ indicating that those with severe MDs were less likely to be born in Austria (82% vs. 94%).

**Table 1:** Sample characteristics

	No MDs (*n* = 778)		At least one MD (*n* = 229)		Diff †	Non-severe MDs (*n* = 112)		Severe MDs (*n* = 117)		Diff †
	*n* Mean	% (SD)	*n* Mean	% (SD)	*p*-value	*n* Mean	% (SD)	*n* Mean	% (SD)	*p*-value
Age*	47	(13)	45	(13)		45	(13)	46	(14)	
Female gender	381	49%	128	56%	0.068	57	51%	71	61%	0.136
Place of living										
< 5000	115	15%	13	6%		5	4%	8	7%	
5000–49,999	358	46%	66	29%		31	28%	35	30%	
50,000–499,999	180	23%	62	27%		34	30%	28	24%	
> 500,000 (Vienna)	125	16%	88	38%	**0.000**	42	38%	46	39%	0.662
Born in Austria	689	89%	201	88%	0.780	105	94%	96	82%	**0.007**
Education										
Compulsory school	58	7%	29	13%		11	10%	18	15%	
Apprenticeship training	185	24%	59	26%		35	31%	24	21%	
Technical college	110	14%	25	11%		11	10%	14	12%	
High school	184	24%	58	25%		26	23%	32	27%	
University	235	30%	58	25%	0.066	29	26%	29	25%	0.329
Missing	6	1%	0	0%		0	0%	0	0%	
Current employment status										
Employed full-time	425	55%	80	35%		46	41%	34	29%	
Employed part-time	69	9%	32	14%		14	13%	18	15%	
Self-employed	89	11%	20	9%		11	10%	9	8%	
Regular retirement	73	9%	15	7%		5	4%	10	9%	
Early retirement due to ill health	45	6%	27	12%		10	9%	17	15%	
Unemployed	24	3%	28	12%		13	12%	15	13%	
Other	53	7%	27	12%	**0.000**	13	12%	14	12%	0.417
Socio-economic status										
High class	87	11%	13	6%		7	6%	6	5%	
Middle class	241	31%	56	24%		28	25%	28	24%	
Lower-middle class	286	37%	65	28%		34	30%	31	26%	
Upper-lower class	127	16%	76	33%		33	29%	43	37%	
Lower class	27	3%	17	7%	**0.000**	9	8%	8	7%	0.830
Missing	10	1%	2	1%		1	1%	1	1%	
Living situation										
Living alone	151	19%	63	28%		29	26%	34	29%	
Living with a partner	281	36%	70	31%		34	30%	36	31%	
Living with children without a partner	56	7%	20	9%		9	8%	11	9%	
Living with children and partner	208	27%	49	21%		25	22%	24	21%	
Living with relatives	55	7%	13	6%		6	5%	7	6%	
Other	25	3%	14	6%	**0.026**	9	8%	5	4%	0.812
Missing	2	0%	0	0%		0	0%	0	0%	
Number of people in household (incl. children)										
1	157	20%	66	29%		30	27%	36	31%	
2	320	41%	92	40%		49	44%	43	37%	
3	141	18%	30	13%		15	13%	15	13%	
≥ 4	150	19%	37	16%	**0.025**	16	14%	21	18%	0.630
Missing	10	1%	4	2%		2	2%	2	2%	

*p*-values ≤ 0.05 are marked in bold

*MD* mental disorder, *SD* standard deviation, *Diff* difference

^†^Chi-square test

^*^t-test

Half (50%) of the respondents with at least one MD, met criteria for two or more mental health diagnoses. Respondents with MDs had more physical comorbidities, compared to those without MDs (Online Resource Table 1). Over one-quarter (28%) of the respondents with at least one MD reported four or more physical comorbidities, compared to 12% in the no MDs group.

### Resource use

The level of missing values was below 1.5% for all resource use categories.

The similar proportion of the respondents with and without MDs had at least one GP (82% vs. 80%) or non-mental health specialist (89% vs. 90%) contacts in the last 12 months (Table 2). However, the mean number of GP and non-mental health specialist contacts per year was significantly higher for the respondents with MDs indicating more frequent use of these services (Table 3). In terms of the mental health specific services use, and use of antidepressants or other psychiatric medication, significant difference was observed between respondents with MDs and without MDs, and between respondents with severe MDs and non-severe MDs (Table 3). Among those with at least one MD, 37% had any resource use of outpatient mental health specific services; 51% of those with severe MDs and 23% of those with non-severe MDs (Table 2).

**Table 2:** Participants with any resource use in the past 12 months

	No MDs (*n* = 778)		At least one MD (*n* = 229)		Diff †	Non-severe MDs (*n* = 112)		Severe MDs (*n* = 117)		Diff †
Variables	*n*	%	*n*	%	*p*-value	*n*	%	*n*	%	*p*-value
*Outpatient non-mental health specific*										
GP	620	80%	189	82%	0.342	89	79%	100	85%	0.231
Specialist physician	698	90%	204	89%	0.783	97	87%	107	91%	0.240
Non-physician^a^	267	34%	94	41%	0.062	40	36%	54	46%	0.108
Medical check-up	314	41%	79	35%	0.105	33	30%	46	40%	0.117
*Outpatient mental health specific*	60	8%	86	37%	0.000	26	23%	60	51%	0.000
Psychiatrist	14	2%	41	18%	**0.000**	8	7%	33	28%	**0.000**
Psychotherapist	35	4%	51	22%	**0.000**	16	14%	35	30%	**0.007**
Psychologist	12	1%	14	6%	**0.000**	7	6%	7	6%	
Other mental health services^b^	11	1%	23	10%	**0.000**	4	4%	19	16%	**0.002**
*Inpatient*										
Non-mental health	117	15%	35	15%	0.927	16	14%	19	16%	0.717
Mental health	2	0%	7	3%		0	0%	7	6%	
*Medication*										
Antidepressants^c^	35	4%	66	29%	**0.000**	22	20%	44	38%	**0.046**
Other medication from group *N*^d^	97	12%	71	31%	**0.000**	28	25%	43	37%	0.064
*Lost productivity*										
Informal care last year	11	1%	13	6%	**0.000**	6	5%	7	6%	0.931
Absence from work last year	378	49%	96	42%	0.077	50	45%	46	4%	0.566
Unemployed last year	37	5%	38	17%	**0.000**	18	16%	20	17%	0.790
Early retirement last year	45	6%	27	12%	**0.002**	10	9%	17	14%	0.189

*p*-values ≤ 0.05 are marked in bold

*MD* mental disorder, *Diff* difference

^†^Chi-square test

^a^Non-physician category includes: ergotherapist, physiotherapist, nurse, social worker, life counselling

^b^Other mental health services include: counselling centre, psychiatric day clinic, psychosocial care, sheltered workshop

^c^Medication from group Nervous system according to the WHO Anatomical Therapeutic Chemical (ATC) classification system (ATC code in parentheses): antidepressants (N06A) and psycholeptics and psychoanaleptics in combination (N06C)

^d^Medication from group Nervous system according to the WHO Anatomical Therapeutic Chemical (ATC) classification system (ATC code in parentheses): antipsychotics (N05A), anxiolytics (N05B), hypnotics and sedatives (N05C), opioids (N02A), other analgesics and antipyretics (N02B), antiepileptics (N03A), anticholinergic agents (N04A)

**Table 3:** Observed health and social care resource use in the past 12 months

	No MDs (*n* = 778)		At least one MD (*n* = 229)		Diff †	Non-severe MDs (*n* = 112)		Severe MDs (*n* = 117)		Diff †
Variables	*n*	Mean (SD)	*n*	Mean (SD)	*p*-value	*n*	Mean (SD)	*n*	Mean (SD)	*p*-value
*Outpatient (contacts) non-mental health specific*										
GP	771	2.82 (3.97)	223	4.50 (6.38)	**0.000**	109	3.58 (6.22)	114	5.39 (6.42)	**0.005**
Specialist physician	758	4.97 (6.37)	221	7.16 (10.27)	**0.002**	106	5.76 (8.39)	115	8.44 (11.63)	**0.022**
Non-physician^a^	774	5.02 (0.55)	224	6.52 (0.99)	0.116	110	4.18 (0.79)	114	8.77 (19.02)	0.115
Medical check-up	770	0.41 (0.49)	227	0.35 (0.48)	0.105	111	0.30 (0.46)	116	0.40 (0.49)	0.117
*Outpatient (contacts) mental health specific*										
Psychiatrist	777	0.08 (0.83)	227	1.25 (4.70)	**0.000**	112	0.74 (4.90)	115	1.75 (4.47)	**0.000**
Psychotherapist	774	0.35 (2.30)	225	3.16 (8.74)	**0.000**	110	1.27 (5.45)	115	4.96 (10.73)	**0.002**
Psychologist	778	0.11 (1.18)	227	0.55 (4.06)	**0.001**	110	0.11 (0.05)	117	0.97 (0.52)	0.589
Other mental health services^b^	778	0.08 (0.89)	229	3.75 (23.96)	**0.000**	112	0.56 (4.81)	117	6.79 (32.97)	**0.001**
*Inpatient (days)*										
Non-mental health specific	774	1.10 (5.05)	229	1.30 (5.30)	0.380	112	1.05 (4.00)	117	1.53 (6.32)	0.503
Mental health specific	778	0.05 (0.90)	228	1.11 (8.20)	**0.000**	112	0	116	2.18 (11.42)	

*p*-values ≤ 0.05 are marked in bold

*MD* mental disorder, *SD* standard deviation, *Diff* difference

^†^Wilcoxon rank-sum (Mann–Whitney) test

^a^Non-physician category includes: ergotherapist, physiotherapist, nurse, social worker, life counselling

^b^Other mental health services include: counselling centre, psychiatric day clinic, psychosocial care, sheltered workshop

Participants with MDs had significantly more weeks of unemployment and months of early retirement due to health problems in the past year (Table 4). The difference in days of absence from work was not statistically significant between participants with MDs vs. no MDs.

**Table 4:** Lost productivity in the last 12 months

	No MDs (*n* = 778)		At least one MD (*n* = 229)		Diff †	Non-severe MDs (*n* = 112)		Severe MDs (*n* = 117)		Diff †
Variables	*n*	Mean (SD)	*n*	Mean (SD)	*p*-value	*n*	Mean (SD)	*n*	Mean (SD)	*p*-value
Informal care (hours per week)	365	0.43 (4.94)	110	1.94 (11.75)	**0.000**	52	0.94 (3.40)	58	2.82 (15.87)	0.906
Absence from work (days)	667	9.74 (32.69)	194	10.23 (31.99)	0.207	97	7.47 (27.05)	97	12.98 (36.19)	0.915
Unemployed (weeks)	773	0.90 (5.49)	227	5.62 (14.62)	**0.000**	112	5.10 (13.63)	115	6.11 (15.57)	0.766
Early retirement (months)	777	0.64 (2.67)	229	1.23 (3.55)	**0.003**	112	1.07 (3.44)	116	1.38 (3.66)	0.289

*p*-values ≤ 0.05 are marked in bold

*MD* mental disorder, *SD* standard deviation, *Diff* difference

^†^Wilcoxon rank-sum (Mann–Whitney) test

### Differences by cost categories

**Fig. 1:**
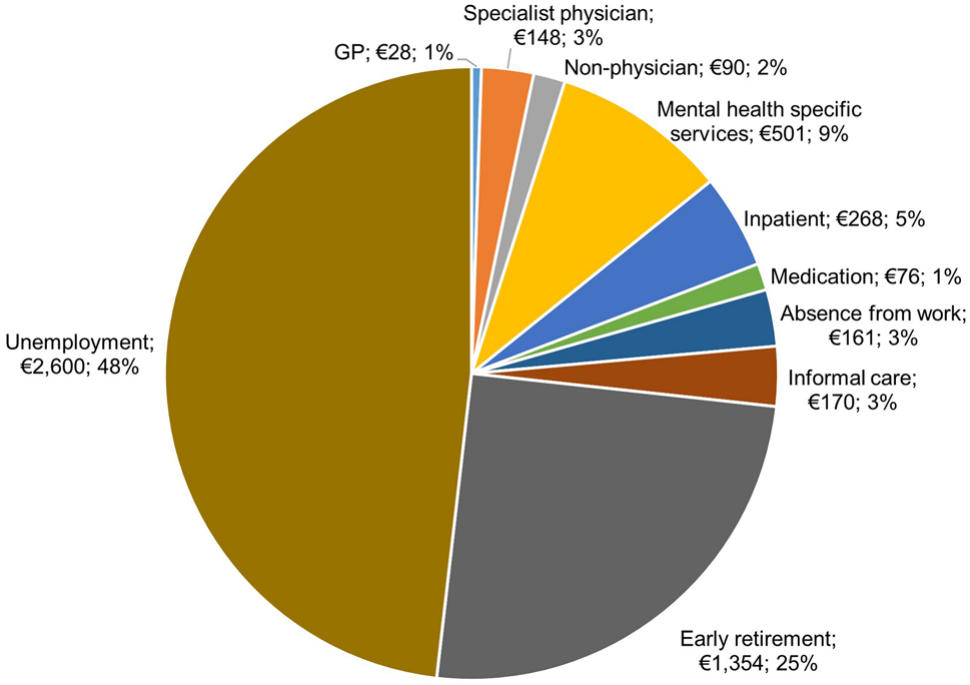
Distribution of the difference in the total mean annual (unadjusted) cost between respondents with MDs and respondents without MDs by cost categories. Mental health specific services include costs for: psychiatrist, psychotherapist and other mental health services (i.e. counselling centre, psychiatric day clinic, psychosocial care, sheltered workshop); non-physician category includes costs for: ergotherapist, physiotherapist, nurse, social worker, life counselling

The difference in the total mean annual (unadjusted) cost between the two groups is shown in Fig. 1. The mean annual cost of GP and specialist physician was higher for respondents with MDs by €28 (standard error, SE: 6) and €148 (SE: 53). The highest cost difference between the two groups was observed for the unemployment and early retirement costs. The mean annual additional cost for the uptake of mental health services for the respondents suffering from MDs was €501 (SE: 54) per year. Mean cost values for all cost categories are showed in Online Resource Table 3.

### Excess cost of mental disorders

The total excess cost (including health and social care, medication and productivity loss costs) of having at least one MD was twice as high compared to no MDs (exp(ß) 2.03 SE: 0.29; *p* < 0.001) in the analysis adjusting for age, sex, education level and number of physical comorbidities (Table 5). The cost for health and social care was 37% higher among people with MDs compared to the respondents without MDs, albeit not reaching statistical significance at 5% level (*p* = 0.06). Excess costs of GP contacts (€19; SE: 7), outpatient mental health specific services (€727, SE: 267) and medication (€151, SE: 76) were statistically significantly higher for those with MDs. Respondents suffering from severe MDs had over 2.5-times higher health and social care cost (exp(ß) 2.86 SE: 0.82; *p* < 0.001) and 9-times higher outpatient mental health specific costs (exp(ß) 8.90; SE: 5.68; *p* = 0.001) compared to non-severe MDs.

**Table 5:** 12-month excess costs of MDs (in EUR 2016)

	MDs vs. no MDs			Severe MDs vs. non-severe MDs		
Variables	Excess cost (SE)^b^	exp (ß) †	95% CI	Excess cost (SE)^b^	exp (ß) †	95% CI
Health and social care	€788 (468)	1.37	0.98–1.92	€3,649 (1,531)	2.86***	**1.64–5.01**
Outpatient (contacts) non-mental health specific	€84 (97)	1.10	0.89–1.36	€365 (204)	1.45*	**1.02–2.06**
GP	€19 (7)	1.42**	**1.13–1.77**	€30 (15)	1.64*	**1.11–2.41**
Specialist physician	€77 (49)	1.20	0.96–1.48	€141 (93)	1.36	0.95–1.94
Non-physician^a^	€2.70 (67)	1.01	0.70–1.44	€187 (157)	1.55	0.84–2.83
Medical check-up	€-6 (3)	0.81*	**0.66–1.00**	€11 (7)	1.38	0.97–1.98
Outpatient (contacts) mental health specific^b^	€727 (267)	19.17***	**9.42–39.01**	€556 (336)	8.90***	**2.55–31.08**
Inpatient (days)	€160 (456)	1.13	0.59–2.15	€4,428 (3,025)	5.04**	**1.74–14.58**
Medication	€151 (76)	2.35**	**1.29–4.29**	€127 (126)	1.98	0.69–5.56
Lost productivity	€4,937 (1,320)	2.63***	**1.86–3.74**	€1,838 (1,644)	1.47	0.81–2.68
Total cost	€5,411 (1,360)	2.03***	**1.55–2.67**	€4,285 (2,246)	1.73*	**1.09–2.74**

Excess cost estimated using predictive mean values

*MD* mental disorder, *SE* standard error

^†^Exponentiated coefficients estimated using GLM with link log and family gamma adjusting for sex, age, education level and number of physical comorbidities

*p ≤ 0.05, **p ≤ 0.01, ***p ≤ 0.001

^a^Non-physician category includes costs for: ergotherapist, physiotherapist, nurse, social worker, life counselling

^b^Outpatient (contacts) mental health specific includes costs for: psychiatrist, psychotherapist, psychologist, counselling centre, psychiatric day clinic, psychosocial care, sheltered workshop

The difference in inpatient cost was not statistically significant between respondents with MDs compared to those without MDs. However, this cost difference was significant between the participants with severe MDs compared to non-severe MDs (exp(ß) 5.04 SE: 2.73; *p* = 0.003). This difference was attributed mostly to the cost of inpatient psychiatric stay, which was reported only within the severe MDs group and was an infrequent event (*n* = 7).

Cost of lost productivity was over 2.5-times higher among people with MDs compared to those without MDs (exp(ß) 2.63; SE: 0.47; *p* < 0.001). Although, lost productivity cost was higher among the severe MDs group compared to non-severe MDs, this difference was not statistically significant (*p* = 0.21).

Results of an exploratory disease-specific cost analysis are provided in Online Resource Table 4. The groups F2 (Schizophrenia, schizotypal and delusional disorders) and F5 (Behavioural syndromes associated with physiological disturbances and physical factors) had sample sizes of *n* = 10 and *n* = 15 only, and there was a high level of uncertainty around the point estimates for all disease categories allowing no reliable analysis results. No statistically significant association between the disease categories and any of the sociodemographic factors could be observed.

### Impact of physical comorbidities on excess cost

The association between physical comorbidities and cost of lost productivity was weaker compared to the association between physical comorbidities and health and social care cost and medication cost (Table 6). Presence of one or two physical comorbidities was not statistically significantly associated with the cost of lost productivity, whereas presence of at least one physical comorbidity was a significant determinant of the health and social care and medication costs.

**Table 6:** Demographic characteristics and number of physical comorbidities as predictors of costs

	Health and social care cost				Lost productivity cost				Medication cost				Total cost			
	Model 1		Model 2		Model 1		Model 2		Model 1		Model 2		Model 1		Model 2	
	exp(ß)†	(95% CI)	exp(ß)†	(95% CI)	exp(ß)†	(95% CI)	exp(ß)†	(95% CI)	exp(ß)†	(95% CI)	exp(ß)†	(95% CI)	exp(ß)†	(95% CI)	exp(ß)†	(95% CI)
Mental health status																
No MDs (ref)																
At least one MD	1.49*	**(1.05–2.11)**	1.37	(0.98–1.92)	2.49***	**(1.73–3.59)**	2.63***	**(1.86–3.74)**	2.05*	**(1.09–3.87)**	2.35**	**(1.29–4.29)**	2.02***	**(1.52–2.68)**	2.03***	**(1.55–2.67)**
Sex																
Men (ref)																
Women	0.88	(0.66–1.18)	0.87	(0.65–1.17)	0.89	(0.66–1.20)	0.87	(0.65–1.16)	0.85	(0.48–1.52)	0.89	(0.52–1.51)	0.90	(0.71–1.15)	0.91	(0.72–1.14)
Age group																
18–24 (ref)																
25–34	0.98	(0.50–1.93)	1.05	(0.55–2.00)	1.36	(0.65–2.83)	1.20	(0.60–2.42)	0.62	(0.18–2.19)	0.88	(0.27–2.84)	1.12	(0.64–1.96)	1.08	(0.63–1.85)
35–44	1.21	(0.60–2.45)	1.00	(0.51–1.98)	1.43	(0.68–2.99)	1.02	(0.50–2.11)	2.20	(0.70–9.66)	2.85	(0.86–9.41)	1.34	(0.76–2.38)	1.04	(0.59–1.81)
45–54	2.11*	**(1.09–4.10)**	1.64	(0.86–3.13)	2.00	(0.98–4.09)	1.54	(0.77–3.08)	4.30*	**(1.33–13.90)**	5.01**	**(1.66–15.15)**	2.04*	**(1.18–3.53)**	1.61	(0.94–2.72)
55–65	1.65	(0.87–3.12)	1.43	(0.77–2.64)	4.62***	**(1.81–7.23)**	2.67**	**(1.36–5.24)**	4.32*	**(1.37–13.59)**	5.25**	**(1.79–15.39)**	2.60***	**(1.53–4.42)**	2.04**	**(1.22–3.41)**
Physical comorbidities																
No comorbidities (ref)																
1 comorbidity			1.49*	**(1.01–2.22)**			1.03	(0.69–1.55)			3.58***	**(1.70–7.52)**			1.26	(0.92–1.74)
2 comorbidities			2.31***	**(1.51–3.54)**			1.19	(0.76–1.86)			3.65***	**(1.71–7.82)**			1.67**	**(1.18–2.35)**
3 comorbidities			3.01***	**(1.91–4.74)**			2.13**	**(1.32–3.43)**			4.01***	**(1.78–9.03)**			2.39***	**(1.65–3.47)**
≥ 4 comorbidities			4.02***	**(2.45–6.49)**			2.30***	**(1.43–3.69)**			7.81***	**(3.45–17.65)**			3.98***	**(2.04–4.32)**

All models were adjusted for education level

*MD* mental disorder, *SE* standard error

^†^Exponentiated coefficients estimated using GLM with link log and family gamma

*p ≤ 0.05, **p ≤ 0.01, ***p ≤ 0.001

### Sensitivity analyses

In our sample the age groups ‘18–24’ and ‘35–44’ were under-represented and the age group ‘55–65’ was over-represented, compared to the official population statistics for the year 2016 [57]. In terms of sex, men constituted 49.45% of the study sample, compared to 50.27% in the general population [57]. Sensitivity analysis results applying sampling post-stratification weights in our cost calculations are provided in Online Resource. In the weighted excess cost analysis, all result conclusions remained unchanged except for health and social care costs which became statistically significantly higher for the respondents with MDs vs. those without MDs (exp(ß) 1.46 SE: 0.26; *p* = 0.04) (Online Resource Table 5). The impact of physical comorbidities remained unchanged compared to the base case analysis (Online Resource Table 6).

In a separate complete cases analysis conducted on the observed data, the significance of the excess cost of MDs remained the same except for the difference in total cost between severe and non-severe MDs which lost statistical significance (Results available from the corresponding author on request).

## Discussion

### Summary of key findings

This study looked at the excess cost associated with MDs based on a cross-sectional population-based survey in Austria. Among the non-institutionalised adult population, the 12-month prevalence of MDs was 22%. The mean annual total cost of health and social care and lost productivity was twice as high among respondents with MDs compared to those without MDs confirming and quantifying the significant economic burden associated with MDs in Austria.

The study showed that while similar proportion of the respondents with and without MDs had at least one GP or non-mental health specialist contacts in the past 12-month period, those with MDs had more frequent contacts. These results suggest that access to healthcare services in general is similar in both groups; however, due to the presence of mental and physical comorbidities respondents with MDs utilise also more non-mental health specific services.

A subgroup analysis comparing those with severe MDs to those with non-severe MDs revealed further significant cost differences between these two groups. Respondents with severe MDs had 1.4-times higher outpatient non-mental health specific costs, 9-times higher cost of outpatient mental health specific services, and over 5-times higher inpatient cost compared to non-severe MDs group.

### Policy considerations

The approach adopted in the current study that looks at MDs in general instead of focusing on individual mental health diagnoses provides supporting information for future resource allocation decisions for mental health related services. Since most mental health services are not designed for one specific disease group but rather can be accessed by people with most mental health diagnoses, the study gives realistic estimates about the overall resource utilisation needs of people with MDs. Approximately half of those suffering from MDs in our sample had multiple mental health diagnoses which means that they would likely utilise more diverse services.

Although results of the present study suggest that access to healthcare services is widespread in Austria, community-based mental health specific services (e.g., psycho-social services, day centres) were rather rarely utilised by the survey respondents with MDs. Only 10% of the respondents with MDs had any resource use in this category suggesting that access to these type of services needs improvement. Use of outpatient mental health specific services, including contacts with psychiatrists, psychotherapists and psychologist and other mental health specific care, was reported by 37% of those with diagnoses of MDs. If we add respondents who also reported psychiatric medication use according to the WHO ATC (anatomic–therapeutic classification) system, listed in the legends to Table 2, this percentage increased to 52%. This correction still leaves around half of those with a MD without using professional help for mental health problems in the past year suggesting diagnostic, availability, access or stigma related service limitations. Furthermore, this finding highlights that using administrative healthcare data for estimating the prevalence of MDs is likely to lead to substantial underestimation of the number of affected people. For example, a report of the SHI from 2009 provides an estimation of the adult population being affected by MDs in Austria based on insurance claim data at around 900,000 (11%) [58]. This estimate is half of that found in the current survey (22%).

The difference in health and social care costs between those with severe MDs and non-severe MDs was statistically significant, whereas the difference in the cost of productivity loss was less prominent and not statistically significant between the two groups. This suggests that also those with less severe MDs are at an increased risk for unemployment and early retirement and labour market participation is significantly affected by the occurrence of any MD. These findings further support the need for prevention and early intervention services available for mentally ill people that target MDs at onset or during less severe phases, to reduce the large excess cost of MDs attributable to lost productivity. Recent evidence suggests significant positive effect of the return-to-work (RTW) programmes among people with MDs [59, 60]

### Effect of physical comorbidities on the excess cost

The effect of physical comorbidities in cost-of-illness studies has been widely debated [61, 62]. Failing to control for comorbidities leads to obtaining increased incremental cost of the investigated disease and causes double-counting of costs [62, 63]. However, adjusting for comorbidities that are caused by the disease of interest would lead to underestimation of the cost estimates associated with the disease [61]. In the current study, the excess cost associated with MDs was estimated while adjusting for the number of physical comorbidities. When costs were adjusted for the number of physical comorbidities, the excess health and social care cost estimate associated with MDs was reduced. On the other hand, estimates of excess lost productivity cost and medication cost increased. One way to interpret these results is that the effect of physical comorbidities is greater on the health and social care costs than on the cost of loss productivity.

### Comparison with other studies

Findings from this study show similar patterns of resource use in the health and social care sectors and similar costs associated with MDs compared to the previously published Austrian literature. One previous study examined access points to different levels of care in Austria based on cross-sectional data from the Austrian Health Interview Survey (ATHIS) from the year 2006–07 [64]. The study reported similar frequency of GP visits (78.8% vs. 80% in the current study), specialist and outpatient visits (86% vs. 90% in the current study), and inpatient hospital stay (22.8% vs. 15% in the current study) [64]. International comparisons of the utilisation of the services data are difficult due to the varying definitions of services and differences in the organisation of mental healthcare services between countries. For example, 21.7% of people with any mental disorder used services provided by mental health professionals (psychiatrist, psychologist or other non-psychiatrist mental health professional or use of a mental health hotline), in the US [65]. In Sweden, utilisation of specialised outpatient mental healthcare was found among 18.7%, 26.0%, 30.1%, and 36.6% patients with sub-syndromal, mild, moderate and severe depression, respectively [66]. In the other studies, the percentage of people with MDs having contact with professional care in an outpatient setting in a 1-year period was 34% in the Netherlands [67], 35% in Australia [68], and 50% in Canada [69]. The present study provides a comparable estimate of 37% of the study respondents with at least one MD who used mental health specific outpatient care.

Evidence about the proportion of the total cost attributed to the cost of loss productivity available in the literature is diverse. In the current study, 69% of the total cost was attributed to lost productivity for those diagnosed with MDs. In the study from the US, 62% of total economic burden of depression was associated with workplace costs [70]. In the Netherlands, 85% of total cost of nine common MDs was associated with productivity losses [33]. Another study from Sweden reported that 65% of total cost of depression was related to productivity loss [71].

### Limitations

Resource use measurement in our study did not include visits to accident and emergency rooms or stay at the intensive care unit. Information about the use of outpatient care (specialist visits) was collected together with visits in single physician’s office and home visits. Moreover, out-of-pocket expenditure and family cost (e.g., travel to healthcare facilities) were not measured. While these simplifications may have implications on the accuracy of the current cost estimates and may lead to some underestimates in the absolute costs, they are unlikely to impact the overall findings and conclusions.

Secondly, the survey did not include any clinical measures of disease severity. Therefore, the distinction between severe and non-severe MDs was made based on the type of diagnosis. While this is an accepted categorisation method, this can introduce some distortion in the interpretation of our severity categorisation. On the other hand, the limited sample size did not allow for a robust cost analysis at ICD-10 disease group category levels. Any conclusion drawn from the results of this exploratory analysis (Online supplement Table 4) should be treated with caution.

Thirdly, the study sample design was based on private home addresses [34]. Therefore, homeless people and people in (long-term) institutional care such as nursing homes, sheltered housing, long-term patients of hospital wards, people in prisons and other closed settings were not subject to investigation. According to the literature, roughly 70% of prison population and homeless population might suffer from MDs [72, 73], and 20% of older adults might suffer from dimensional depression [74]. In Austria, up to 70% of people residing nursing homes and in prisons exhibit symptoms of MDs [75]. Therefore, the current cost estimates are likely to be an underestimation at full population level and should be seen as representative of the general non-institutionalised adult (non-elderly) population in Austria but not the whole Austrian population.

Finally, the sampling process conducted for this study was based on the distribution of sex, age and population size in each Austrian federal state. Post-sampling weights were used to adjust for any observed distribution differences in a sensitivity analysis. No information in terms of the socio-economics status was available for those who declined to participate in the study in the first place. When comparing the characteristics of the study sample to the available national statistics, we found good representativeness in terms of employment status. The proportion of employed or self-employed persons in the sample was 71% compared to 71.5% employment rate in 2017 in Austria, while the unemployment rate in the study sample was 5.16% compared to 7.1% in the national statistics [76]. In terms of education level, we found that the proportion of people with higher education was somewhat over-represented and the population of those with lower level of education was somewhat under-represented in the study. According to the national statistics, 13% of Austrians completed tertiary education and 26% accomplished compulsory school compared to 29% and 9% in our study, respectively. This difference could be partially related to the under-representation of the younger age groups in this study.

## Conclusions

This study provided first estimates of the excess economic burden of MDs in Austria based on general population survey-based data. The findings show that in comparison to the population without MDs, the burden posed on the health and social care sectors was found higher for people with MDs, especially for severe MDs. MDs contribute to substantially higher lost productivity costs through both unemployment and early retirement. This study provides information necessary for future mental health policies which should strengthen community-based care and provision of mental healthcare services targeting improvement in participation in the labour market among those suffering from MDs. Future studies should examine utilisation of services and resulting costs of specific MDs given existing comorbidities and across bigger cohorts (including institutionalised or difficult to reach groups) for fully comprehensive cost estimates.

## Supplementary Information

Below is the link to the electronic supplementary material.

Supplementary file1 (DOCX 47 KB)
