# Supplementary material for: The excess economic burden of mental disorders: findings from a cross-sectional prevalence survey in Austria
Source: Eur J Health Econ. 2020 May 26;21(7):1075–89. doi: 10.1007/s10198-020-01200-0 (PMC7423789; doi:10.1007/s10198-020-01200-0)
Supplement: Supplementary file 1 — Supplementary file1 (DOCX 47 KB) [file 10198_2020_1200_MOESM1_ESM.docx]

**Electronic Supplementary Material**

**Table 1**. Self-reported comorbidities in the last 12 months

|  | **No MDs**  **(n=778)** | | **At least one MD**  **(n=229)** | | **Non-severe MDs (n=112)** | | **Severe MDs (n=117)** | |
| --- | --- | --- | --- | --- | --- | --- | --- | --- |
|  | n | % | n | % | n | % | n | % |
| **Physical comorbidities** |  |  |  |  |  |  |  |  |
| High blood pressure | 129 | 17% | 56 | 24% | 26 | 23% | 30 | 26% |
| Cardio-vascular diseases | 98 | 13% | 44 | 19% | 23 | 21% | 21 | 18% |
| Asthma | 33 | 4% | 19 | 8% | 9 | 8% | 10 | 9% |
| Chronic bronchitis | 34 | 4% | 17 | 7% | 7 | 6% | 10 | 9% |
| Gastritis or gastric ulcers | 47 | 6% | 31 | 14% | 13 | 12% | 18 | 15% |
| Gallbladder inflammation or gallstones | 17 | 2% | 5 | 2% | 4 | 4% | 1 | 1% |
| Liver diseases | 2 | 0% | 4 | 2% | 0 | 0% | 4 | 3% |
| Disorders of thyroid gland | 62 | 8% | 28 | 12% | 14 | 13% | 14 | 12% |
| Diabetes | 30 | 4% | 14 | 6% | 7 | 6% | 7 | 6% |
| High blood cholesterol | 134 | 17% | 56 | 24% | 24 | 21% | 32 | 27% |
| Gout or high level of uric acid | 21 | 3% | 16 | 7% | 7 | 6% | 9 | 8% |
| Anaemia | 33 | 4% | 19 | 8% | 8 | 7% | 11 | 9% |
| Kidney diseases | 10 | 1% | 6 | 3% | 1 | 1% | 5 | 4% |
| Cancer | 15 | 2% | 3 | 1% | 2 | 2% | 1 | 1% |
| Osteoarthritis | 194 | 25% | 72 | 31% | 37 | 33% | 35 | 30% |
| Inflammatory joint or spinal disease | 50 | 6% | 17 | 7% | 6 | 5% | 1 | 1% |
| Osteoporosis | 25 | 3% | 13 | 6% | 2 | 2% | 11 | 9% |
| Epilepsy | 3 | 0% | 0 | 0% | 0 | 0% | 0 | 0% |
| Multiple sclerosis | 5 | 1% | 1 | 0% | 0 | 0% | 1 | 1% |
| Migraine | 66 | 8% | 39 | 17% | 20 | 18% | 19 | 16% |
| Allergies | 237 | 30% | 109 | 48% | 29 | 26% | 50 | 43% |
| Other | 69 | 9% | 20 | 9% | 9 | 8% | 11 | 9% |
| **Number of physical comorbidities** |  |  |  |  |  |  |  |  |
| 0 | 201 | 26% | 41 | 18% | 19 | 17% | 22 | 19% |
| 1 | 223 | 29% | 36 | 16% | 21 | 19% | 15 | 13% |
| 2 | 133 | 17% | 61 | 27% | 29 | 26% | 32 | 27% |
| 3 | 128 | 16% | 26 | 11% | 13 | 12% | 13 | 11% |
| ≥4 | 93 | 12% | 65 | 28% | 30 | 27% | 35 | 30% |

Note: MD – mental disorder

## **Table 2.** Unit costs for resource use valuation in euro (€) 2016

| **Resource use** | **Valuation (€)** | **Unit** | **Source** |
| --- | --- | --- | --- |
| **Outpatient non-mental health specific** |  |  |  |
| GP* | 15.9 | per contact | Ärztekosten-Jahresstatistik, 2015 ^2^ |
| Internist* | 115.7 | per contact ^1^ |  |
| Gynaecologist* | 56.6 | per contact ^1^ |  |
| Ophthalmologist* | 57.0 | per contact ^1^ |  |
| Orthopaedist* | 89.0 | per contact ^1^ |  |
| ENT* | 66.7 | per contact ^1^ |  |
| Neurologist* | 97.2 | per contact ^1^ |  |
| Surgeon* | 130.3 | per contact ^1^ |  |
| Dermatologist* | 46.4 | per contact ^1^ |  |
| Urologist* | 68.2 | per contact ^1^ |  |
| Radiologist* | 87.7 | per contact ^1^ |  |
| Doctor for homeopathy* | 182.5 | per contact | Market price (range €120.00- €250.00) [1] |
| Other alternative medicine* | 108.5 | per contact | Market price (range €80.00- €140.00) [2] |
| Occupational medicine* | 137.8 | per contact | Assumption one contact = one hour; Österreichische Ärztekammer: Honorarempfehlung Arbeitsmedizin, 2015 [3] |
| Other specialist (not specified)^3^* | 45.1 | per contact | Ärztekosten-Jahresstatistik, 2015 ^2^ |
| Ergotherapist | 70.0 | per contact | Zechmeister et al. 2011 [4] |
| Physiotherapist* | 74.0 | per contact | Market price (60 minutes assumed) [5] |
| Other therapist (e.g. dance therapy)* | 59.2 | per contact | Market price for ‘Kunsttherapeutische Einzelsitzung’ [6] |
| Nurse* | 31.7 | per contact | Assumed ‘Beratungsgespräch Krankenpflege (30 min)’ https://www.ris.bka.gv.at/Dokumente/LgblAuth/LGBLA_WI_20140129_2/Ambulatoriumsbeitraege_Anlage.pdfsig |
| Social worker | 13.3 | per contact | https://www.gehaltskompass.at/ ^4^ |
| Life consulter | 65.0 | per contact | Expert opinion (range €50.00-€80.00) [7] |
| Naturopath | 75.0 | per contact | Market price [8] |
| Other health professionals (not specified) | 70.0 | per contact | Ergotherapist assumed |
| Medical check-up* | 72.3 | per contact | Reimbursed tariffs from social insurance funds per health check-up ^5^ |
| **Outpatient mental health specific** |  |  |  |
| Psychiatrist* | 108.8 | per contact ^1^ | Ärztekosten-Jahresstatistik, 2015 ^2^ |
| Psychotherapist | 71.8 | per contact | Reimbursement a 60-minute session from the social insurance fund €21.80 [9] + an average patient co-payment of €50 [10] |
| Psychologist | 40.5 | per contact | Cost per test (45 minutes) https://www.psychologenforum.at/pdf/VPsyAktuell/GesamtvertragKompiliert2010_ZV5.pdf |
| Psychiatric day clinic* | 35.8 | per contact | Own calculations ^6^ |
| Counselling Centre | 35.8 | per session | Assumption = psychiatric day clinic |
| Socio-psychiatric services* | 60.1 | per contact | Assumed ‘Sozialpsychiatrische Intervention und/oder Psychoedukation’ https://www.psychologenforum.at/pdf/VPsyAktuell/GesamtvertragKompiliert2010_ZV5.pdf |
| Sheltered workshops* | 35.8 | per service | Assumption = psychiatric day clinic |
| Other (not specified) | 71.8 | per session | Assumption = psychotherapy |
| **Inpatient** |  |  |  |
| Internal medicine | 622.0 | per day ^7^ | Ministry of Labour, Social Affairs, Health and Consumer Protection (BMASGK), 2018, unpublished data |
| Gynaecology | 1,034.0 | per day ^7^ |  |
| Ophthalmology | 853.0 | per day ^7^ |  |
| Surgery | 905.0 | per day ^7^ |  |
| Orthopaedics | 811.0 | per day ^7^ |  |
| ENT | 871.0 | per day ^7^ |  |
| Neurology | 592.0 | per day ^7^ |  |
| Psychiatry | 419.0 | per day ^7^ |  |
| Psychosomatics | 302.0 | per day ^7^ |  |
| Dermatology | 729.0 | per day ^7^ |  |
| Urology | 856.0 | per day ^7^ |  |
| Other | 767.0 | per day ^7^ |  |
| **Medication** | various | per milligram | Erstatungskodex [11] |
| **Lost productivity** |  |  |  |
| Informal care | 11.03 | per hour | https://www.gehaltskompass.at/ ^8^ |
| Mean gross salary for part-time work | 11.5 | per hour | Statistik Austria [12] |
| Mean gross salary for full-time work | 14.8 | per hour | Statistik Austria [12] |

Note:

*Costs deflated or inflated to 2016;

^1^ Value per quarter (= per consultation based on the assumption of 1 consultation per quarter);

^2^ The latest version of Ärztekosten-Jahresstatistik that is available online is from year 2013 [13]. The document with statistics for year 2015 was received from the Orgaisation of Austrian Social Security (Hauptverband der österreichischen Sozialversicherungsträger) upon a phone request;

^3^ Assumed the average cost per consultation with a specialist (Allgemeine Fachärzte) based on Ärztekosten-Jahresstatistik, 2015

^4^ Assumption 60 min duration of a contact; wage per hour was calculated based on the average monthly gross salary for social workers ‘SozialarbeiterIn’ €2,610 (range € 2,470-€ 2,750), 40 hours per week;

^5^ Average tariff based on catalogues from various SHI funds, 2015, e.g. <http://www.bgkk.at/cdscontent/load?contentid=10008.615425&version=1425301890> , <http://www.sgkk.at/cdscontent/load?contentid=10008.603401&version=1421763802>;

^6^ Calculations based on the report performed for day psychiatric services in Vienna (2014) “Kuratorium für Psychosoziale Dienste in Wien, Prüfung des Bereiches Arbeit und Beschäftigung/Tagesstruktur“. The total cost (€2,653,460) was divided by the total number of services provided (€76,936) and inflated to 2016 <http://www.stadtrechnungshof.wien.at/berichte/2016/lang/02-21-StRH-II-PSD-1-15.pdf> ;

^7^ Cost calculated by dividing the total cost per hospital department by a sum of midnight stays and number of one-day cases;

^8^ Wage per hour was calculated based on the average monthly gross salary for a housekeeper ‘HaushälterIn’ €1,765 (range € 1,670 – 1,860/month), 40 hours per week.

**Table 3.** Imputed unadjusted 12-month mean costs in EUR 2016

|  | **No MDs**  **(n=778)** | | **At least one MD**  **(n=229)** | | **Difference MDs vs. no MDs^*^** | **Non-severe MDs (n=112)** | | **Severe MDs (n=117)** | | **Difference Severe vs. non-severe MDs^*^** |
| --- | --- | --- | --- | --- | --- | --- | --- | --- | --- | --- |
|  | **Mean** | **SE** | **Mean** | **SE** |  | **Mean** | **SE** | **Mean** | **SE** |  |
| **Health and social care total** | 2,068 | 185 | 3,100 | 403 | 1,032† | 1,824 | 358 | 4,321 | 695 | 2,497‡ |
| Outpatient (contacts) non-mental health specific | 797 | 46 | 1,060 | 98 | 263‡ | 805 | 94 | 1,303 | 167 | 498† |
| GP | 45 | 2 | 73 | 7 | 28‡ | 58 | 10 | 87 | 10 | 29† |
| Specialist physician | 377 | 24 | 525 | 51 | 148‡ | 412 | 52 | 634 | 85 | 222† |
| Non-physician^1^ | 346 | 33 | 436 | 60 | 90 | 313 | 58 | 553 | 102 | 240† |
| Medical check up | 29 | 1 | 25 | 2 | -4 | 21 | 3 | 29 | 3 | 8 |
| Outpatient (contacts) mental health specific | 42 | 8 | 543 | 96 | 501‡ | 196 | 99 | 875 | 158 | 679‡ |
| Psychiatrist | 9 | 3 | 136 | 34 | 127‡ | 81 | 50 | 189 | 45 | 108 |
| Psychotherapist | 25 | 6 | 225 | 41 | 200‡ | 91 | 37 | 353 | 71 | 262‡ |
| Psychologist | 5 | 2 | 22 | 11 | 17† | 5 | 4 | 39 | 21 | 34 |
| Other mental health services^2^ | 3 | 1 | 160 | 63 | 157‡ | 20 | 16 | 294 | 121 | 274† |
| Inpatient | 1,229 | 165 | 1,497 | 337 | 268 | 823 | 286 | 2,142 | 595 | 1,318† |
| Non-mental health specific | 1,210 | 163 | 1,032 | 256 | -178 | 823 | 286 | 1,231 | 420 | 408 |
| Mental health specific | 19 | 14 | 465 | 228 | 446‡ | 0 | 0 | 910 | 442 |  |
| **Medication** | 108 | 24 | 184 | 25 | 76 | 152 | 35 | 215 | 37 | 63 |
| **Lost productivity** | 3,003 | 264 | 7,288 | 736 | 4,285‡ | 6,198 | 995 | 8,332 | 1,077 | 2,134 |
| Informal care | 47 | 21 | 217 | 75 | 170‡ | 241 | 127 | 193 | 81 | -48 |
| Absence from work | 1,001 | 130 | 1,162 | 283 | 161 | 790 | 303 | 1,518 | 470 | 728 |
| Early pension | 1,451 | 213 | 2,805 | 525 | 1,354‡ | 2,357 | 714 | 3,234 | 767 | 877 |
| Unemployment | 504 | 111 | 3,104 | 536 | 2,600‡ | 2,809 | 708 | 3,387 | 803 | 578 |
| **Total cost** | 5,179 | 373 | 10,572 | 908 | 5,393‡ | 8,174 | 1,147 | 12,868 | 1,369 | 4,694‡ |

Note: † significant at 5% level; ‡ significant at 1% level; **^*^** Values derived from univariable OLS

^1^ Non-physician category includes: ergotherapist, physiotherapist, nurse, social worker, life counselling

^2^ Other mental health services include: counselling centre, psychiatric day clinic, psychosocial care, sheltered workshop

**Table 4.** 12-month excess costs per disease category of MDs (in EUR 2016), analysis on imputed data

|  | **Health and social care** | | **Medication** | | **Lost productivity** | | **Total cost** | |
| --- | --- | --- | --- | --- | --- | --- | --- | --- |
|  | **exp(ß)**† | **(95% CI)** | **exp(ß)**† | **(95% CI)** | **exp(ß)**† | **(95% CI)** | **exp(ß)**† | **(95% CI)** |
| F1 | 0.96 | (0.51-1.82) | 1.35 | (0.37-4.92) | 2.43** | (1.28-4.61) | 1.72* | (1.04-2.85) |
| F2 | 5.10* | (1.33-19.60) | 5.93 | (0.51-69.51) | 3.33 | (0.88-12.67) | 3.83* | (1.31-11.23) |
| F3 | 1.82* | (1.11-2.98) | 2.03 | (0.86-4.81) | 1.91** | (1.19-3.07) | 1.85** | (1.27-2.70) |
| F4 | 0.74 | (0.47-1.15) | 1.85 | (0.76-4.49) | 1.76** | (1.16-2.68) | 1.28 | (0.92-1.79) |
| F5 | 1.29 | (0.55-3.05) | 0.37 | (0.08-1.68) | 0.73 | (0.31-1.71) | 0.93 | (0.47-1.83) |

Note: MD – mental disorder; F1 - Disorders due to psychoactive substance use, F2 – Schizophrenia, schizotypal and delusional disorders, F3 - Mood [affective] disorders, F4 - Neurotic, stress-related and somatoform disorders, F5 – Behavioural syndromes associated with physiological disturbances and physical factors

† exponentiated coefficients estimated using GLM with link log and family gamma adjusting for sex, age, education level and number of physical comorbidities

*p≤0.05, **p≤0.01, ***p≤0.001

**Table 5.** 12-month excess costs of MDs (in EUR 2016), analysis on weighted data

|  | **At least one MD vs. no MDs** | | | **Severe MDs vs. non-severe MDs** | | |
| --- | --- | --- | --- | --- | --- | --- |
|  | **Excess cost (SE)^3^** | **exp (ß)** † | **95% CI** | **Excess cost (SE)^2^** | **exp (ß)** † | **95% CI** |
| **Health and social care** | €917 (512) | **1.46*** | **1.01-2.11** | €3,702 (1526) | **2.97***** | **1.70-5.19** |
| Outpatient (contacts) non-mental health specific | €13 (96) | 1.02 | 0.81-1.27 | €340 (191) | **1.43*** | **1.02-2.02** |
| GP | €16 (8) | **1.35*** | **1.04-1.74** | €25 (15) | **1.55*** | **1.01-2.38** |
| Specialist physician | €78 (47) | 1.21 | 0.98-1.49 | €114 (93) | 1.30 | 0.88-1.92 |
| Non-physician^1^ | €-53 (68) | 0.86 | 0.59-1.26 | €237 (153) | 1.68 | 0.99-2.87 |
| Medical check-up | €-8 (3) | **0.72*** | **0.54-0.95** | €5 (8) | 1.20 | 0.71-2.01 |
| Outpatient (contacts) mental health specific^2^ | €760 (228) | **19.09***** | **10.55-34.55** | €558 (244) | **8.42***** | **3.34-21.19** |
| Inpatient (days) | €1,269 (918) | 2.08 | 0.98-4.42 | €3,898 (2,936) | **4.54*** | **1.38-15.00** |
| **Medication** | €161 (59) | **2.61***** | **1.57-4.31** | €24 (70) | 1.17 | 0.48-2.85 |
| **Lost productivity** | €3,860 (1,085) | **2.51***** | **1.74-3.65** | €2,331 (1,561) | 1.76 | 0.96-3.21 |
| **Total cost** | €4,867 (1,341) | **2.06***** | **1.51-2.80** | €4,778 (2,313) | **1.94**** | **1.19-3.17** |

Note: MD – mental disorder; SE – standard error; † exponentiated coefficients estimated using GLM with link log and family gamma adjusting for sex, age, education level and number of physical comorbidities;

^1^ Non-physician category includes: ergotherapist, physiotherapist, nurse, social worker, life counselling

^2^ Outpatient (contacts) mental health specific includes: psychiatrist, psychotherapist, psychologist, counselling centre, psychiatric day clinic, psychosocial care, sheltered workshop

^3^ Excess cost estimated using predictive mean values; p-values: *p≤0.05, **p≤0.01, ***p≤0.001;

**Table 6.** Demographic characteristics and number of physical comorbidities as predictors of costs (weighted analysis).

|  | **Health and social care cost** | | | | **Lost productivity cost** | | | | **Medication cost** | | | | **Total cost** | | | |
| --- | --- | --- | --- | --- | --- | --- | --- | --- | --- | --- | --- | --- | --- | --- | --- | --- |
|  | **Model 2** | | **Model 1** | | **Model 2** | | **Model 1** | | **Model 2** | | **Model 1** | | **Model 2** | | **Model 1** | |
|  | **exp(ß)**† | **(95% CI)** | **exp(ß)**† | **(95% CI)** | **exp(ß)**† | **(95% CI)** | **exp(ß)**† | **(95% CI)** | **exp(ß)**† | **(95% CI)** | **exp(ß)**† | **(95% CI)** | **exp(ß)**† | **(95% CI)** | **exp(ß)**† | **(95% CI)** |
| **Mental health status** |  |  |  |  |  |  |  |  |  |  |  |  |  |  |  |  |
| No MDs (ref) |  |  |  |  |  |  |  |  |  |  |  |  |  |  |  |  |
| At least one MD | **1.65*** | **(1.12-2.45)** | **1.45*** | **(1.02-2.08)** | **2.34***** | **(1.61-3.38)** | **2.55***** | **(1.76-3.70)** | **2.39**** | **(1.43-3.97)** | **2.81***** | **(1.73-4.60)** | **2.03***** | **(1.48-2.79)** | **2.05***** | **(1.51-2.77)** |
| **Sex** |  |  |  |  |  |  |  |  |  |  |  |  |  |  |  |  |
| Men (ref) |  |  |  |  |  |  |  |  |  |  |  |  |  |  |  |  |
| Women | 0.93 | (0.67-1.30) | 0.99 | (0.75-1.32) | 0.93 | (0.66-1.32) | 0.91 | (0.66-1.25) | 0.88 | (0.55-1.40) | 0.84 | (0.54-1.32) | 0.94 | (0.72-1.24) | 0.97 | (0.76-1.25) |
| **Age group** |  |  |  |  |  |  |  |  |  |  |  |  |  |  |  |  |
| 18-24 (ref) |  |  |  |  |  |  |  |  |  |  |  |  |  |  |  |  |
| 25-34 | 1.31 | (0.67-2.55) | 1.31 | (0.78-2.20) | 1.64 | (0.76-3.52) | 1.39 | (0.66-2.93) | 0.89 | (0.18-2.19) | 1.11 | (0.44-2.83) | 1.42 | (0.79-2.56) | 1.32 | (0.77-2.26) |
| 35–44 | 1.55 | (0.80-3.02) | 1.16 | (0.69-1.95) | 1.61 | (0.70-3.67) | 1.07 | (0.49-2.32) | **3.50*** | **(0.70-9.66)** | **3.95*** | **(1.49-10.47)** | 1.60 | (0.88-2.91) | 1.16 | (0.68-2.01) |
| 45–54 | **2.74**** | **(1.43-5.24)** | **1.86*** | **(1.09-3.18)** | **2.24*** | **(1.05-4.80)** | 1.63 | (0.77-3.45) | **6.77**** | **(1.33-13.90)** | **6.50***** | **(2.53-16.70)** | **2.42**** | **(1.38-4.25)** | **1.77*** | **(1.04-3.03)** |
| 55–65 | **1.89*** | **(1.02-3.53)** | 1.56 | (0.95-2.54) | **3.76***** | **(1.83-7.72)** | **2.56*** | **(1.22-5.39)** | **6.39***** | **(1.37-13.59)** | **6.16***** | **(2.58-14.71)** | **2.84***** | **(1.66-4.87)** | **2.07**** | **(1.23-3.47)** |
| **Physical comorbidities** |  |  |  |  |  |  |  |  |  |  |  |  |  |  |  |  |
| No comorbidities (ref) |  |  |  |  |  |  |  |  |  |  |  |  |  |  |  |  |
| 1 comorbidity |  |  | **1.62*** | **(1.11-2.38)** |  |  | 1.11 | (0.70-1.74) |  |  | **3.00**** | **(1.43-3.29)** |  |  | 1.37 | (0.97-1.94) |
| 2 comorbidities |  |  | **2.84***** | **(1.75-4.58)** |  |  | 1.35 | (0.82-2.24) |  |  | **3.59***** | **(1.84-6.99)** |  |  | **1.97**** | **(1.32-2.93)** |
| 3 comorbidities |  |  | **2.88***** | **(1.92-4.34)** |  |  | **2.61**** | **(1.47-4.63)** |  |  | **3.83***** | **(2.04-7.19)** |  |  | **2.71***** | **(1.82-4.04)** |
| ≥4 comorbidities |  |  | **4.92***** | **(3.21-7.54)** |  |  | **2.59***** | **(1.57-4.26)** |  |  | **7.73***** | **(3.52-16.98)** |  |  | **3.48***** | **(2.42-5.01)** |
|  |  |  |  |  |  |  |  |  |  |  |  |  |  |  |  |  |
|  |  |  |  |  |  |  |  |  |  |  |  |  |  |  |  |  |

Note: MD – mental disorder; SE – standard error; † exponentiated coefficients estimated using GLM with link log and family gamma; All models were adjusted for education level

# **References**

1. Österreichische Geselschaft für Homöopathische Medizin. http://www.homoeopathie.at/faqs_patienten/wie-hoch-sind-die-kosten-einer-behandlung-und-welche-leistungen-kann-ich-ueber-die-krankenkasse-zurueckerstattet-bekommen/. Accessed 19 May 2019

2. Zentrum für Integrative Medizin. http://www.integrative-medizin.at/de/aerzte,20533,33905.html. Accessed 19 May 2019

3. Österreichische Ärztekammer: Honorarempfehlung Arbeitsmedizin. http://www.aekwien.at/documents/4771581/21872017/Honorarempfehlung+Arbeitsmedizin+2015.pdf/99450697-87ca-42a8-9be5-cc0558d5c34e?version=1.1&t=1481719171000 (2015). Accessed 19 May 2019

4. Zechmeister, I., Loibl, T.: Eltern-Kind-Vorsorge neu. Teil III: Ist-Erhebung der Finanzierungs-und Kostenstrukturen von Eltern-Kind Leistungen in Österreich. Ludwig Boltzmann Institut für Health Technology Assessment, Wien (2011)

5. Physio Aspern: Kosten & Refundierung. http://www.physio-aspern.at/kosten.html# (2017). Accessed 19 May 2019

6. Kunsttherapie-praxis: Preise. http://www.kunsttherapie-praxis.at/kontakt/preise/ (2017). Accessed 19 May 2019

7. Mag. Franz Landerl, D.: Berufsgruppensprecher der Lebens- und SozialberaterInnen in der WKO Oberösterreich. http://www.nachrichten.at/freizeit/guides/lebensberatung/Lebensberatung-Alles-was-Sie-darueber-wissen-muessen;art132682,2028179 (2016).

8. Praxis naturheilung: Preise 2016. https://www.praxis-natur-heilung.at/ueber-die-praxis/kosten/ (2016). Accessed 19 May 2019

9. Bundesministerium für Gesundheit und Frauen: Psychotherapie; p. 32. http://www.bmgf.gv.at/cms/home/attachments/2/2/6/CH1452/CMS1148314652459/psychotherapie30102012.pdf. Accessed 19 May 2019

10. psyonline.at. http://www.psyonline.at/contents/7437/ueberblick-kosten-der-psychotherapie. Accessed 19 May 2019

11. Hauptverband der österreichischen Sozialversicherungsträger: Erstatungskodex - EKO. http://www.hauptverband.at/cdscontent/?contentid=10007.693708&viewmode=content (2016). Accessed 19 May 2019

12. Statistik Austria: Bruttostunden-, Bruttomonats- und Bruttojahresverdienste nach Wirtschaftstätigkeit 2014. http://www.statistik.at/web_de/statistiken/menschen_und_gesellschaft/soziales/personen-einkommen/verdienststruktur/index.html (2017). Accessed February 20, 2019

13. Hauptverband der österreichischen sozialversicherungsträger: Ärztekosten-Jahresstatistik. https://www.parlament.gv.at/PAKT/VHG/XXV/AB/AB_06216/imfname_480326.pdf. Accessed February 20, 2019
